# Dietary supplementation for female infertility: Recent advances in the nutritional therapy for premature ovarian insufficiency

**DOI:** 10.3389/fmicb.2022.1001209

**Published:** 2022-11-17

**Authors:** Qixin Han, Zi-Jiang Chen, Yanzhi Du

**Affiliations:** ^1^Center for Reproductive Medicine, Renji Hospital, School of Medicine, Shanghai Jiao Tong University, Shanghai, China; ^2^Shanghai Key Laboratory for Assisted Reproduction and Reproductive Genetics, Shanghai, China; ^3^Center for Reproductive Medicine, Cheeloo College of Medicine, Shandong University, Jinan, China; ^4^National Research Center for Assisted Reproductive Technology and Reproductive Genetics, Shandong University, Jinan, China; ^5^Key Laboratory of Reproductive Endocrinology of Ministry of Education, Shandong University, Jinan, China

**Keywords:** premature ovarian insufficiency, female fitness, nutrition, diet, microbiota, menopause

## Abstract

Premature ovarian insufficiency (POI) ranks top in the reproductive disorders that may impair multiple functioning systems, reduce the quality of life and ultimately deprive patients of their fertility among women. Symptoms can be partially alleviated by present hormone replacement therapy that cannot improve conception or decrease occurrence rates of systemic complication. Nutritional dietary supplements are attracting more and more attention because of their safety, bioavailability, and efficacy for well-being. Nutrients in the daily food are composed of carbohydrates, fat and lipoprotein, protein and polypeptide, vitamins, and vegetable or fruits containing phytoestrogens. These are functional nutrients due to the proliferative, anti-inflammatory, anti-oxidant, and mitochondria-protective potential during the course of menopause. Apart from dietary nutrients, microbe-related nutritional substances, including probiotics, prebiotics and the combination-synbiotics, display high potential as well in supporting estrous cycle, ovarian viability and modulating other vital reproductive functions. The present review will discuss dietary and microbial nutrients and their roles and applications in the living body based upon animal or human research, evaluate possible effect mechanisms from molecular, cellular and tissue levels, and provide insights into nutritional therapy for prolonging reproductive lifespan in female patients.

## Introduction

Premature ovarian insufficiency (POI), previously termed as premature ovarian failure, is a common and severe clinical reproductive disorder among women (Hsieh and Ho, 2021). It is primarily characterized by the decrease or loss of ovarian function occurring before the age of 40, with a high incidence of 1% (Ford et al., 2020). Its main clinical manifestations include menstrual disorders (amenorrhea or irregular menstruation), increased pituitary gonadotropin levels, decreased estrogen levels and other perimenopausal changes, especially the decrease in fertility (Di-Battista et al., 2020). In addition, POI patients also have mood changes, hot flashes, sleep disturbances, bone pain and pruritus, and experience long-term harm to the cardiovascular system, skeletal motor system, nervous system and mental health that all together affect women’s fertility (Horton et al., 2019). At present, the only effective treatment for POI is hormone replacement therapy (HRT). Though HRT can alleviate symptoms of low estrogen, rebuild regular menstrual cycle, and lower blood follicle stimulating hormone levels, this therapy may bring potential risks, such as the occurrence of thromboembolic disease, and tumor. Moreover, it hardly slows down the pathological process of ovarian senescence and functional decline, not to mention improving the conception rate of POI patients (European Society for Human Reproduction and Embryology (ESHRE) Guideline Group on POI, Webber et al., 2016). In conclusion, the insufficient understanding of the mechanism of POI leads to the failure of current treatment methods for the effective delay of ovarian function decline. Consequently, it is of great significance to explore the key pathogenic factors involved in the process of POI to improve the efficacy of POI clinical treatment.

**FIGURE 1:**
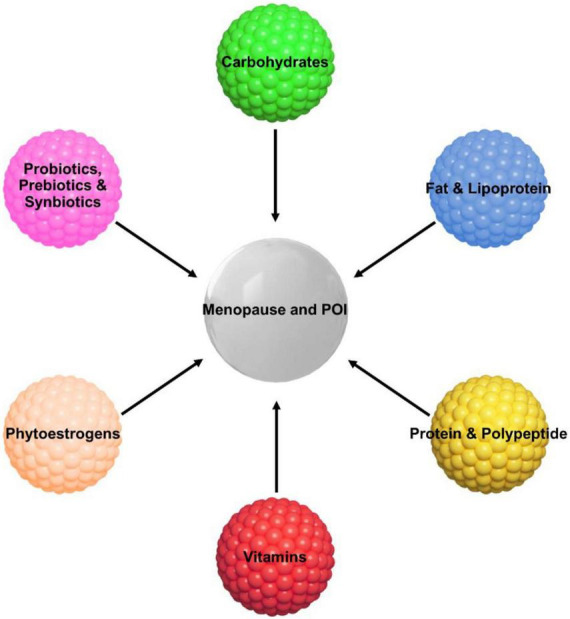
Primary nutritional factors for progression, alleviation and control of menopause and POI.

In the past decades, the highly increasing incidence of POI is accompanied with the rapid modification in the living lifestyle and deterioration of environment (Golezar et al., 2020). This arouses interest and concerns in the influence of the environmental and life factors on reproductive health, in addition to those genetic, immune or iatrogenic factors (Qin et al., 2015; Golezar et al., 2020). Furthermore, among all these factors, nutrient intake in daily diet of lifestyle is the most accessible and modifiable for POI patients, and remains the least understandable of all factors (Chen et al., 2017). The dietary habit was changed long ago with the improvement in socioeconomic levels worldwide. However, it also brings malnutrition issues to both genders, young and old due to obesity, emaciation, and different types of eating disorders. There are a few studies about the nutrient status and regulation affecting female fertility and menopause (Fontana and Della Torre, 2016; Chiu et al., 2018; Silvestris et al., 2019). Herein, we preliminarily propose the primary dietary factors and nutrients for disease progression, alleviation and control in menopausal course of POI, and expect these considerations may shed light on future clinical protection and treatment of ovarian function (Figure 1 and Table 1).

**TABLE 1:** Representatives from different nutritional components and their potential effects on menopause and POI.

Nutritional component	Specific substance	Effect	References
Carbohydrates	Galactose	Harmful	Rostami Dovom et al., 2019
	Polysaccharides from dendrobium officinal	Beneficial	Wu et al., 2018
Fat and lipoprotein	Triglycerides	Harmful	Knauff et al., 2008
	High-density lipoprotein	Uncertain	Berg et al., 2004; Knauff et al., 2008
	Total cholesterol	Harmful	Gulhan et al., 2012
	Low-density lipoprotein	Harmful	Gulhan et al., 2012
	Polyunsaturated fat	Harmful	Sapre and Thakur, 2014
	Total fat and saturated fat	No Influence	Sapre and Thakur, 2014
Protein and polypeptide	Proteins from sea food and fresh eggs	Beneficial	Wang et al., 2018
	Oyster polypeptides	Beneficial	Li et al., 2020
Vitamins	Niacin	Beneficial	Asadi et al., 2021
	Folic acid	Beneficial	Szymański and Kazdepka-Ziemińska, 2003; Cueto et al., 2016
	MTHFR	Beneficial	Goyco Ortiz et al., 2019
	Vitamin C	Beneficial	Hou et al., 2020
	Vitamin D	Uncertain	Kebapcilar et al., 2013; Ersoy et al., 2016
	Vitamin E	Beneficial	Ma et al., 2021; Safiyeh et al., 2021
Phytoestrogens	Soy isoflavone	Beneficial	Yoneda et al., 2011; Meléndez et al., 2015
	Sesame seed lignans	Beneficial	Kinugasa et al., 2011
	Sesamol	Beneficial	Kaur et al., 2013
Probiotics, prebiotics, and synbiotics	*Lactobacillus reuteri*	Beneficial	Britton et al., 2014
	*Lactobacilli* and cranberry extract complex	Beneficial	Koradia et al., 2019
	chitosan and citrus pectin	Beneficial	Wu et al., 2021
	*Lactobacillus paracasei* and inulin	Beneficial	Timan et al., 2014
	*Lactobacillus fermentum* and β-glucan	Beneficial	Jeong et al., 2017

## Carbohydrates

Carbohydrates provide the most abundant life-supporting energy source for all living creatures. They are typically classified into monosaccharide, oligosaccharide, and polysaccharide (Kikkeri et al., 2010). Galactose is a kind of commonly seen monosaccharides reported in the POI and related infertile diseases (Bandyopadhyay et al., 2003). Dovom et al. claimed that it exerted a toxicity effect on ovaries based on systematic review analysis (Rostami Dovom et al., 2019). Therefore, galactose may be applied for induction of POI animal model under necessary concentration, appropriate time and exposure. Galactose may also inhibit follicular migration toward the gonadal region and lead to decreased ovarian reserves and estradiol synthesis (Rostami Dovom et al., 2019). Therefore, abnormal sugar galactose metabolism is related with POI onset and progression.

Polysaccharides from dendrobium officinal (DO) induced a protective effect on POI process in natural aging rodents (Wu et al., 2018). They were administered at 70 mg/kg through oral route and helped these rats regain normal weight and alleviate pathological changes to the ovary, such as vessel proliferation reduction and follicular decrease. DO-oriented polysaccharides reduced inflammatory insults in the ovarian tissues by regulating the NF-κB and p53/Bcl-2 signaling pathways and upregulated activities of glutathione peroxidase (GSH-Px) and superoxide dismutase (SOD) and decreased malondialdehyde (MDA) concentration in the serum to exert anti-oxidative effects. At the same time, they also manipulated the mitochondrial function and restored the ovarian cell viability (Wu et al., 2018). The significance of carbohydrates for POI onset or progression should be evaluated further.

## Fat and lipoprotein

Fat and high body weight may be relevant to control of POI severity. Several studies reported early menopause was associated with low body mass index (Tao et al., 2015; Szegda et al., 2017). The exact mechanisms are unclear. Lipoproteins are transportable forms of fat that actively participate in metabolic activities in the human body (Hoeke et al., 2016). Knauff et al. (2008) performed lipid profiles in POI patients and identified higher triglycerides (TG) concentration and moderately lower high-density lipoprotein (HDL) level in the serum were correlated with free androgen index increase and sex hormone binding globulin loss. These findings indicated that a TG increase might lead to an insulin sensitivity decrease (Knauff et al., 2008). In another retrospective clinical study, Gulhan et al. (2012) similarly found that young female patients who presented POI syndromes without any previous hormone intake history showed higher total cholesterol (TC) and low-density lipoprotein (LDL) concentrations in the serum in comparison with normal females of close epidemiological features. The results implicated that undesirable lipid alteration was triggered under deprived estrogen conditions in female POI patients (Gulhan et al., 2012). However, the concentration level of HDL is very inconsistent among different studies, with some reporting elevated levels, and others showing decreased levels in menopause women (Berg et al., 2004). As to the impact of fat intake on menopause, differences may be caused due to the various kinds of fat, including polyunsaturated, total and saturated fat. Sapre and Thakur (2014) reported that high consumption of polyunsaturated fats was related to earlier menopause onset; in the meantime, total fat and saturated fat intake barely influenced menopausal period and age. Nevertheless, it is unclear whether different fat types in the daily diet may affect POI pathogenesis in a certain way.

## Protein and polypeptide

High protein intake was reported to be useful to postpone the early arrival of menopause (Taneja et al., 2019). Wang et al. (2018) found that consumption of seafood (around 3 days/week) and fresh eggs (more than 4 days/week) was positively correlated with late menopausal onset. Several other researches also supported similar conclusions. Dorjgochoo et al. (2008) claimed that high protein consumption was beneficial to delay menopause and prolong the reproductive function. The European Prospective Investigation into Cancer and Nutrition (EPIC) also conducted a cohort study, in which early menopausal occurrence was inversely associated with protein intake level (Gold et al., 2013). In addition to direct protein consumption, polypeptide is also significant for protecting ovarian function and delaying ovarian aging (Wang et al., 2021). Li et al. (2020) reported that oyster polypeptides, purified from oyster via enzymolysis, were able to protect against oxidative stress and inflammation to exert a therapeutic effect on POI because of their DPPH (2,2-diphenyl-1-picrylhydrazyl) radical-scavenger ability. Oyster polypeptides could correct the abnormal estrous cycle, and enhance serum follicle stimulating hormone (FSH) and luteinizing hormone (LH) concentrations. Further, they increased primordial follicular count and distribution, and significantly alleviated ovarian cell death by mimicking SOD clearance. The ovarian cell protection and survival supported by oyster polypeptides was activated upon regulating the death receptor BCL-2-dependent signaling pathway (Li et al., 2020). Therefore, appropriate protein and peptide intake may be a potential way to affect POI progression and severity.

## Vitamins

Vitamins are a class of micro-organic substances only from food and maintain some vital physiological activities and functions in the human body. They do not provide energy but regulate metabolic process. Many vitamins play a key role in supporting female health, such as vitamin B, C, D, E, and some coenzymes (Chavarro and Schlaff, 2018).

Niacin belongs to the Vitamin B family, metabolizes cellular energy, and directly impacts normal physiology (Ye et al., 2019). In addition to many neurological and dermatitis diseases, niacin also reduced ovarian cell death by inhibiting oxidative stress to improve follicle development (Asadi et al., 2021). Interestingly, niacin was also able to modify POI phenotype and recover female POI mice induced by chemotherapy or radiation stimulation. The authors further confirmed the addition of 10 Mmol niacin could decrease the expression level of follicle arrest marker FOXO3 and increase the expression level of oocyte marker DDX4 to exert the ovarian protective effects (Asadi et al., 2021). Folic acid is a kind of water-soluble vitamin B and is important to protein synthesis, erythrocyte division and growth. A clinical study reported based on a small sample that females of low fertility showed a higher pregnancy rate after receiving 400-μg folic acid consumption for 12 weeks in comparison with placebo treatment (Westphal et al., 2006). Folic acid consumption reduced the pregnancy time for healthy females and improved the quality and maturation of oocytes from a couple of cohort studies (Szymański and Kazdepka-Ziemińska, 2003; Cueto et al., 2016). MTHFR could strongly affect the folate cycle and help a POI patient who simultaneously had non-Hodgkin’s lymphoma and repeat pregnancy losses, successfully deliver a healthy male baby (Goyco Ortiz et al., 2019).

Vitamin C is a natural antioxidant and is actively involved in stem cell fate regulation (DiTroia et al., 2019). It was reported to modify the biological characteristics of human amniotic epithelial cells (hAECs) and significantly stimulated the ovarian marker expression in the ovary tissues from vitamin C-treated hAECs transplanted POI mice. The possible effecting mechanism was the vitamin C dependent paracrine excretion of several important growth factors, such as hepatocyte growth factor and epidermal growth factor (Hou et al., 2020).

Vitamin D belongs to fat-soluble vitamins and is vital for life support by regulating calcium and phosphate metabolism (Bischoff-Ferrari et al., 2005). Its active form, 1,25-dihydroxy vitamin D_3_ is localized in many organs, such as kidney, liver, brain and ovary (Xu et al., 2016). In the female reproductive system, vitamin D can regulate anti-Müllerian hormone (AMH) secretion in the granulosa cells and consequently affect FSH level (Simpson et al., 2020). However, its role in POI pathogenesis is controversial as some claimed it did not influence pathophysiological process of POI, while others reported vitamin D deficiency was correlated with coagulation and was a primary factor for POI due to follicular dysgenesis and aromatase level decrease (Kebapcilar et al., 2013; Ersoy et al., 2016). Neither did previous researches report any confirmative findings on the vitamin D supplementation for POI treatment. It was found that vitamin D intake at 50,000 IU per week, for around 2 months, was irrelevant to pregnant improvement clinically (Aflatoonian et al., 2014). IVF outcome was not improved either by increasing serum vitamin D level in egg recipient patients (Rudick et al., 2014). Therefore, the interpretation of the significance of vitamin D for POI patients remains vague and still need further investigation.

Vitamin E is well-known beneficial factor for reproductive system because it increases ovarian volume and stimulates proliferation primary follicles (Peh et al., 2016). Vitamin E is mainly composed of tocopherols and tocotrienols that are important for free radical clearance under physiological and pathological conditions (Abraham et al., 2019). Deficiency of vitamin E might lead to ovarian cell death and developmental abnormality in a female rodent model (Das and Chowdhury, 1999). In a clinical study, vitamin E was identified to be related with the onset of POI among 40 patients. The active form of vitamin E-α tocopherol concentration was significantly higher in females with normal menstruation than POI patients. Further analysis showed that this could be associated with the decreased expression level of AMH and indicated ovarian reserve capability was compromised in the POI pathogenesis (Das and Chowdhury, 1999; Ma et al., 2021). In another randomized controlled trial study, vitamin E at 400IU was given to POI patients for 3 months and significantly increased antral follicle count and mean ovarian volume, in addition to the improvement in AMH levels (Safiyeh et al., 2021). The vitamin E supplementation was potentially useful to alleviate POI process and barely exerted side effects.

## Phytoestrogens

Phytoestrogens are not indeed part of estrogens but function similarly by providing a high affinity of estrogen receptor β as hormonal supplements (Wei et al., 2017). They typically involve isoflavones from soy, and lignans from sesame, both of which are important and ordinary food types in our daily diet. Several studies confirmed the reduction effect of phytoestrogen consumption on TC, LDL, and plasma lipids among females after menopause (de Kleijn et al., 2002; Kreijkamp-Kaspers et al., 2005). Isoflavone intake from soy consumption also significantly alleviated iliac artery atherosclerosis reduced the size of coronary artery plaques for those females who consumed soy food before and after menopause within 5 years. The beneficial effect was not obvious for those who started to have soy food as daily diet after menopause (Meléndez et al., 2015). Yoneda et al. (2011) noted that soy isoflavone might reduce hot flushes in a menopausal rodent setting by ovariectomy. The effect was based on the production of isoflavan equol by gut microbiota and estrogen-like mechanism (Yoneda et al., 2011). Sesame seed lignans, one of the components in sesame seed oil increased β-oxidation of fatty acids and prohibited cholesterol absorption in a mouse model (Kinugasa et al., 2011). Sesamol is a strong antioxidant and primary component of sesame. It could ameliorate memory, attenuate anxious emotion, and decrease oxidant insults in the central nervous system. In the meantime, sesamol also regulated the lipid constituent in the serum and decreased inflammatory effects by downregulating tumor necrosis factor-α (TNF-α) concentration in the serum in comparison with menopausal rats after ovariectomy (Kaur et al., 2013).

## Probiotics, prebiotics, and synbiotics

In addition to daily nutrient supplemented in the diet, some beneficial living organisms are also getting increasingly attraction due to their potential influence on female health (Han et al., 2021), especially POI (Wang et al., 2020). They are termed as probiotics (Milani et al., 2017). Probiotics promote digestion and absorption of nutrients, improve immune function, maintain the structural balance of intestinal flora, and enhance the antioxidant level of human body. A number of probiotics are applied in food supplement and medical treatment. For female health, a close correlation is proposed between hormone levels and microbiota from either gut or vagina (Anahtar et al., 2018). Gut microbiota were regarded as an alternate metabolic supplier of estrogens in patients with leiomyoma and breast carcinoma, and were mainly enriched in *E. coli*, *Clostridium* sp., *Lactobacillus* sp., and *Bacteroides* (Kwa et al., 2016; Critchley et al., 2020). Considering these phenomena, the regulatory effect of probiotics may also be exerted by modulating gut microbiota in an indirect style through changing metabolic activity and efficiency of several vegetable constituents with estrogenic or antiestrogenic properties, termed as phytoestrogens, such as soy isoflavones, lignans, or urolithins mentioned in the previous section (Seyed Hameed et al., 2020).

Probiotics are involved in the regulation of premenstrual syndrome, urogenital infection and osteoporosis. Estrogen deficiency is a primary reason of osteoporosis related to bone inflammation and resorption after POI (Samad et al., 2020). Previous studies indicate that gut microbiota show a potential influence on bone formation and metabolism. Bone increase was noticed in mice, rats and birds that received prebiotic therapy (Scholz-Ahrens and Schrezenmeir, 2007; Weaver et al., 2011; Yang et al., 2013). Nevertheless, probiotics were less known as to their roles in bone regulation. Probiotic *Lactobacillus reuteri* inhibited upregulation of bone marrow CD4^+^ T-lymphocytes and consequent osteoclast activation *in vitro* and in ovariectomized rodent study. Britton et al. (2014) assumed that *Lactobacillus reuteri* could directly deactivate T cells and inhibit TNF-α production, and indirectly deactivate stromal cells via T cells to reduce osteoclast cytokine formation, and therefore increase bone metabolic activity and reduce bone loss. In a randomized controlled trial consisting of 60 postmenopausal women ranging from 40 to 60 years, probiotics also enhanced the metabolic activity of isoflavones to alleviate urogenital problems such as dry vagina and bad sex experience after menopause for 16-week consecutive treatment. The effect was comparable to low-dose vaginal estrogen, the gold standard therapy for genitourinary syndrome of menopause (Ribeiro et al., 2018). Szulińska et al. (2018) reported in a 12-week randomized clinical trial that multispecies probiotic bacteria at 1 × 10^10^ colony forming units (CFU) concentration affected gut permeability and cardiometabolic parameters, such as lipopolysaccharide levels, insulin, glucose, uric acid, fat mass, subcutaneous fat and insulin-resistant index. Koradia et al. (2019) found that *Lactobacilli* and cranberry extract complex could prevent recurrent urinary tract infections in female patients before or around menopause for consecutive 26 weeks in a pilot clinical study.

Another type of microbiota-related substances are termed prebiotics. Prebiotics are fermented dietary fibers which contain certain modifications, both in the component and/or bioactivity in the intestinal microbiota providing beneficial effects on health status of the human body (Alenaizan et al., 2021). At present, prebiotics mainly include bifidogenic, non-digestible oligosaccharides (especially galactooligosaccharides, inulin and its hydrolytic product oligofructose). Plenty of nutritional prebiotics may facilitate the bioabsorption of calcium, such as caseinophosphopeptides (CPP), proteolytic outcome of casein that together construct soluble composites with calcium (Ilesanmi-Oyelere and Kruger, 2020). Besides, some complex organic acids, like malic or citric acid can add to the percentage of dissolvable composites, bioabsorbable calcium in the intestine. The potential mechanisms of prebiotic-dependent calcium metabolism include that the acid metabolic products, such as lactic, acetic and propionic acid, from bacteria fermented carbohydrates in the colon lowered the regional pH to elevate the luminal level of calcium ions and to increase passive bioabsorption of calcium. Further, electrical charges might be modified via Ca^2+^-H^+^ composites. Therefore, the prebiotics played a protective role in osteoporosis prevention and inhibition after female menopause (Smejkal et al., 2003; de Vrese, 2009). Wu et al. (2021) showed that chitosan and citrus pectin, two kinds of prebiotics, enhanced glucose tolerance, reduced dyslipidemia, and elevated serum arginine, propionate, leucine and butyrate levels. In addition, chitosan was more useful to modulate gut microbiota and consequently to affect the postmenopausal symptoms (Wu et al., 2021).

Some combination attempts of probiotics and prebiotics were also carried out to produce synbiotics (O’Connor et al., 2021). Timan et al. (2014) showed that *Lactobacillus paracasei* and inulin enhanced the bioactive availability of isoflavones that were beneficial for alleviating menopausal issues. Jeong et al. (2017) reported that using synbiotics composed of *Lactobacillus fermentum* and β-glucan from cauliflower mushroom as probiotics and prebiotics could reduce skin temperature of rodent tails and maintain 17β-estradiol level in the serum and uterine index. The synbiotics dependent mechanisms were further validated as they activated hepatic insulin signaling, followed by AMPK phosphorylation in estrogen-deficient rats (Jeong et al., 2017). These studies preliminarily reflected the beneficial roles of probiotics, prebiotics and synbiotics in regulating menopausal syndromes and their primary pathophysiological process. The promising findings will be vital for the investigation into POI pathogenesis and treatment by microbial nutrient strategy.

## Conclusion

Nutrition is vital for female health throughout the lifespan, especially for post-menopause period. Primary nutrients, such as carbohydrates, fat and lipoprotein, protein and polypeptide, vitamins, and phytoestrogens, have displayed huge potential and enjoyable outcomes in the application for menopausal intervention of POI, both in animal and clinical studies. The dietary nutrients may delay the menopause by affecting various aspects, from improving ovarian viability and function directly to counteracting oxidative stress, inflammation, aging and systemic endocrinal disorders. A latest meta-analysis suggested an association between POI and an increased risk of type 2 diabetes mellitus (T2DM) (Anagnostis et al., 2019). There is increasing evidence indicating that the gut microbiota is included in the pathophysiological process of insulin resistance. Thus, it leads to the progression of T2DM via influencing the contents of some vital metabolites. Nevertheless, it remains to be further validated whether modification of gut microbiota in POI patients results in serum metabolites out of balance, which in turn predisposes to menopause manifestation and related health concerns. Moreover, the therapeutic effects were not consistent in all researches and even among different time points in a single research study. This indicates the individual difference is significant and further work should be emphasized on precision nutrition with individual nutrient needs for optimal female reproduction, like POI treatment. Still, microbial nutrition is rarely discussed in POI patients or animals, possibly due to the unclear pathogenesis of POI. Especially, the correlation between probiotics, prebiotics and synbiotics and POI remains poorly investigated. It should also be a major theme to work on in the future. From the present work, dietary and microbial nutrients are important and promising for regulating female reproductive disorder, like POI, and therefore, optimal lifestyle intervention by diet is promising and deserves more efforts and attention to provide better and healthier life for female individuals.

## Author contributions

QH wrote the manuscript, reviewed the literature, and collected the information. YD and Z-JC revised the manuscript. All authors approved the final manuscript.

## References

[B1] Abraham A. Kattoor A. J. Saldeen T. Mehta J. L. (2019). Vitamin E and its anticancer effects. *Crit. Rev. Food Sci. Nutr.* 59 2831–2838. 10.1080/10408398.2018.1474169 29746786

[B2] Aflatoonian A. Arabjahvani F. Eftekhar M. Sayadi M. (2014). Effect of vitamin D insufficiency treatment on fertility outcomes in frozen-thawed embryo transfer cycles: a randomized clinical trial. *Iran J. Reprod. Med.* 12 595–600. 25469131 PMC4248143

[B3] Alenaizan A. Borca C. H. Karunakaran S. C. Kendall A. K. Stubbs G. Schuster G. B. (2021). X-ray fiber diffraction and computational analyses of stacked hexads in supramolecular polymers: insight into self-assembly in water by prospective prebiotic nucleobases. *J. Am. Chem. Soc.* 143 6079–6094. 10.1021/jacs.0c12010 33852800

[B4] Anagnostis P. Christou K. Artzouchaltzi A. M. Gkekas N. K. Kosmidou N. Siolos P. (2019). Early menopause and premature ovarian insufficiency are associated with increased risk of type 2 diabetes: a systematic review and meta-analysis. *Eur. J. Endocrinol.* 180 41–50. 10.1530/EJE-18-0602 30400047

[B5] Anahtar M. N. Gootenberg D. B. Mitchell C. M. Kwon D. S. (2018). Cervicovaginal microbiota and reproductive health: the virtue of simplicity. *Cell Host Microbe* 23 159–168. 10.1016/j.chom.2018.01.013 29447695

[B6] Asadi N. Izadi M. Aflatounian A. Esmaeili-Dehaj M. Rezvani M. E. Hafizi Z. (2021). Chronic niacin administration ameliorates ovulation, histological changes in the ovary and adiponectin concentrations in a rat model of polycystic ovary syndrome. *Reprod. Fertil. Dev.* 33 447–454. 10.1071/RD20306 33751926

[B7] Bandyopadhyay S. Chakrabarti J. Banerjee S. Pal A. K. Goswami S. K. Chakravarty B. N. (2003). Galactose toxicity in the rat as a model for premature ovarian failure: an experimental approach readdressed. *Hum. Reprod.* 18 2031–2038. 10.1093/humrep/deg414 14507817

[B8] Berg G. Mesch V. Boero L. Sayegh F. Prada M. Royer M. (2004). Lipid and lipoprotein profile in menopausal transition. effects of hormones, age and fat distribution. *Horm. Metab. Res.* 36 215–220. 10.1055/s-2004-814450 15114519

[B9] Bischoff-Ferrari H. A. Willett W. C. Wong J. B. Giovannucci E. Dietrich T. Dawson-Hughes B. (2005). Fracture prevention with vitamin D supplementation: a meta-analysis of randomized controlled trials. *JAMA* 293 2257–2264. 10.1001/jama.293.18.2257 15886381

[B10] Britton R. A. Irwin R. Quach D. Schaefer L. Zhang J. Lee T. (2014). Probiotic *L. reuteri* treatment prevents bone loss in a menopausal ovariectomized mouse model. *J. Cell Physiol.* 229 1822–1830. 10.1002/jcp.24636 24677054 PMC4129456

[B11] Chavarro J. E. Schlaff W. D. (2018). Introduction: impact of nutrition on reproduction: an overview. *Fertil. Steril.* 110 557–559. 10.1016/j.fertnstert.2018.07.023 30196937

[B12] Chen H. Cheng R. Xu L. Z. (2017). Correlation between dietary nutrition and premature ovarian failure. *Sichuan Da Xue Xue Bao Yi Xue Ban* 48 575–578. 28752977

[B13] Chiu Y. H. Chavarro J. E. Souter I. (2018). Diet and female fertility: doctor, what should I eat? *Fertil. Steril.* 110 560–569. 10.1016/j.fertnstert.2018.05.027 30196938

[B14] Critchley H. O. D. Babayev E. Bulun S. E. Clark S. Garcia-Grau I. Gregersen P. K. (2020). Menstruation: science and society. *Am. J. Obstet. Gynecol.* 223 624–664. 10.1016/j.ajog.2020.06.004 32707266 PMC7661839

[B15] Cueto H. T. Riis A. H. Hatch E. E. Wise L. A. Rothman K. J. Sørensen H. T. (2016). Folic acid supplementation and fecundability: a Danish prospective cohort study. *Eur. J. Clin. Nutr.* 70 66–71. 10.1038/ejcn.2015.94 26081493

[B16] Das P. Chowdhury M. (1999). Vitamin E-deficiency induced changes in ovary and uterus. *Mol. Cell Biochem.* 198 151–156. 10.1023/a:1006954032164 10497890

[B17] de Kleijn M. J. van der Schouw Y. T. Wilson P. W. Grobbee D. E. Jacques P. F. (2002). Dietary intake of phytoestrogens is associated with a favorable metabolic cardiovascular risk profile in postmenopausal U.S.women: the Framingham study. *J. Nutr.* 132 276–282. 10.1093/jn/132.2.276 11823590

[B18] de Vrese M. (2009). Health benefits of probiotics and prebiotics in women. *Menopause Int.* 15 35–40. 10.1258/mi.2009.009008 19237621

[B19] Di-Battista A. Moysés-Oliveira M. Melaragno M. I. (2020). Genetics of premature ovarian insufficiency and the association with X-autosome translocations. *Reproduction* 160 R55–R64. 10.1530/REP-20-0338 32841156

[B20] DiTroia S. P. Percharde M. Guerquin M. J. Wall E. Collignon E. Ebata K. T. (2019). Maternal vitamin C regulates reprogramming of DNA methylation and germline development. *Nature* 573 271–275. 10.1038/s41586-019-1536-1 31485074 PMC8423347

[B21] Dorjgochoo T. Kallianpur A. Gao Y. T. Cai H. Yang G. Li H. (2008). Dietary and lifestyle predictors of age at natural menopause and reproductive span in the Shanghai Women’s Health Study. *Menopause* 15 924–933. 10.1097/gme.0b013e3181786adc 18600186 PMC2615483

[B22] Ersoy E. Ersoy A. O. Yildirim G. Buyukkagnici U. Tokmak A. Yilmaz N. (2016). Vitamin D levels in patients with premature ovarian failure. *Ginekol. Pol.* 87 32–36. 10.17772/gp/57839 27306466

[B23] European Society for Human Reproduction and Embryology (ESHRE) Guideline Group on POI, Webber L. Davies M. Anderson R. Bartlett J. Braat D. (2016). ESHRE guideline: management of women with premature ovarian insufficiency. *Hum. Reprod.* 31 926–937. 10.1093/humrep/dew027 27008889

[B24] Fontana R. Della Torre S. (2016). The deep correlation between energy metabolism and reproduction: a view on the effects of nutrition for women fertility. *Nutrients* 8:87. 10.3390/nu8020087 26875986 PMC4772050

[B25] Ford E. A. Beckett E. L. Roman S. D. McLaughlin E. A. Sutherland J. M. (2020). Advances in human primordial follicle activation and premature ovarian insufficiency. *Reproduction* 159 R15–R29. 10.1530/REP-19-0201 31376814

[B26] Gold E. B. Crawford S. L. Avis N. E. Crandall C. J. Matthews K. A. Waetjen L. E. (2013). Factors related to age at natural menopause: longitudinal analyses from SWAN. *Am. J. Epidemiol.* 178 70–83. 10.1093/aje/kws421 23788671 PMC3698989

[B27] Golezar S. Keshavarz Z. Ramezani Tehrani F. Ebadi A. (2020). An exploration of factors affecting the quality of life of women with primary ovarian insufficiency: a qualitative study. *BMC Womens Health* 20:163. 10.1186/s12905-020-01029-y 32758224 PMC7405332

[B28] Goyco Ortiz L. E. Servy E. J. Menezo Y. J. R. (2019). A successful treatment with 5 methyltetrahydrofolate of a 677 TT MTHFR woman suffering premature ovarian insufficiency post a NHL (non-Hodgkin’s lymphoma) and RPL (repeat pregnancy losses). *J. Assist. Reprod. Genet.* 36 65–67. 10.1007/s10815-018-1332-0 30406447 PMC6338595

[B29] Gulhan I. Bozkaya G. Uyar I. Oztekin D. Pamuk B. O. Dogan E. (2012). Serum lipid levels in women with premature ovarian failure. *Menopause* 19 1231–1234. 10.1097/gme.0b013e318254102b 22713860

[B30] Han Q. Wang J. Li W. Chen Z. J. Du Y. (2021). Androgen-induced gut dysbiosis disrupts glucolipid metabolism and endocrinal functions in polycystic ovary syndrome. *Microbiome* 9:101. 10.1186/s40168-021-01046-5 33957990 PMC8103748

[B31] Hoeke G. Kooijman S. Boon M. R. Rensen P. C. Berbée J. F. (2016). Role of brown fat in lipoprotein metabolism and atherosclerosis. *Circ. Res.* 118 173–182. 26837747 10.1161/CIRCRESAHA.115.306647

[B32] Horton J. Sterrenburg M. Lane S. Maheshwari A. Li T. C. Cheong Y. (2019). Reproductive, obstetric, and perinatal outcomes of women with adenomyosis and endometriosis: a systematic review and meta-analysis. *Hum. Reprod. Update* 25 592–632. 10.1093/humupd/dmz012 31318420

[B33] Hou S. Ding C. Shen H. Qian C. Zou Q. Lu J. (2020). Vitamin C improves the therapeutic potential of human amniotic epithelial cells in premature ovarian insufficiency disease. *Stem Cell Res. Ther.* 11:159. 10.1186/s13287-020-01666-y 32321569 PMC7178972

[B34] Hsieh Y. T. Ho J. Y. P. (2021). Thyroid autoimmunity is associated with higher risk of premature ovarian insufficiency-a nationwide Health Insurance Research Database study. *Hum. Reprod.* 36 1621–1629. 10.1093/humrep/deab025 33569594

[B35] Ilesanmi-Oyelere B. L. Kruger M. C. (2020). The role of milk components, Pro-, Pre-, and synbiotic foods in calcium absorption and bone health maintenance. *Front. Nutr.* 7:578702. 10.3389/fnut.2020.578702 33072800 PMC7539038

[B36] Jeong S. Y. Kang S. Hua C. S. Ting Z. Park S. (2017). Synbiotic effects of β-glucans from cauliflower mushroom and *Lactobacillus fermentum* on metabolic changes and gut microbiome in estrogen-deficient rats. *Genes Nutr.* 12:31. 10.1186/s12263-017-0585-z 29151980 PMC5679333

[B37] Kaur A. Jindal S. Kaur I. P. Chopra K. (2013). Effect of sesamol on the pathophysiological changes induced by surgical menopause in rodents. *Climacteric* 16 426–437. 10.3109/13697137.2012.696292 23017032

[B38] Kebapcilar A. G. Kulaksizoglu M. Ipekci S. H. Korkmaz H. Kebapcilar L. Akyurek F. (2013). Relationship between mean platelet volume and low-grade systemic coagulation with vitamin D deficiency in primary ovarian insufficiency. *Arch. Gynecol. Obstet.* 288 207–212. 10.1007/s00404-013-2735-x 23377179

[B39] Kikkeri R. Kamena F. Gupta T. Hossain L. H. Boonyarattanakalin S. Gorodyska G. (2010). Ru(II) glycodendrimers as probes to study lectin-carbohydrate interactions and electrochemically measure monosaccharide and oligosaccharide concentrations. *Langmuir* 26 1520–1523. 10.1021/la9038792 20099915

[B40] Kinugasa C. Naemura A. Hyodo K. Nakai Y. Katsuta M. Yamamoto J. (2011). Experimental antithrombotic effects of sesame seed whole grains and extracts. *Blood Coagul. Fibrinolysis* 22 526–531. 10.1097/MBC.0b013e328347b085 21577091

[B41] Knauff E. A. Westerveld H. E. Goverde A. J. Eijkemans M. J. Valkenburg O. van Santbrink E. J. (2008). Lipid profile of women with premature ovarian failure. *Menopause* 15 919–923. 10.1097/gme.0b013e31816b4509 18551082

[B42] Koradia P. Kapadia S. Trivedi Y. Chanchu G. Harper A. (2019). Probiotic and cranberry supplementation for preventing recurrent uncomplicated urinary tract infections in premenopausal women: a controlled pilot study. *Expert Rev. Anti. Infect. Ther.* 17 733–740. 10.1080/14787210.2019.1664287 31516055

[B43] Kreijkamp-Kaspers S. Kok L. Bots M. L. Grobbee D. E. van der Schouw Y. T. (2005). Dietary phytoestrogens and plasma lipids in Dutch postmenopausal women; a cross-sectional study. *Atherosclerosis* 178 95–100. 10.1016/j.atherosclerosis.2004.06.002 15585205

[B44] Kwa M. Plottel C. S. Blaser M. J. Adams S. (2016). The intestinal microbiome and estrogen receptor-positive female Breast cancer. *J. Natl. Cancer Inst.* 108:djw029. 10.1093/jnci/djw029 27107051 PMC5017946

[B45] Li Y. Qiu W. Zhang Z. Han X. Bu G. Meng F. (2020). Oral oyster polypeptides protect ovary against d-galactose-induced premature ovarian failure in C57BL/6 mice. *J. Sci. Food Agric.* 100 92–101. 10.1002/jsfa.9997 31435952

[B46] Ma L. Chen G. Xu W. Chen P. Lan Y. Huang Y. (2021). The relationship between vitamin E level and premature ovarian insufficiency. *J. Obstet. Gynaecol. Res.* 47 1481–1486. 10.1111/jog.14659 33438304

[B47] Meléndez G. C. Register T. C. Appt S. E. Clarkson T. B. Franke A. A. Kaplan J. R. (2015). Beneficial effects of soy supplementation on postmenopausal atherosclerosis are dependent on pretreatment stage of plaque progression. *Menopause* 22 289–296. 10.1097/GME.0000000000000307 25072952 PMC4309745

[B48] Milani C. Duranti S. Bottacini F. Casey E. Turroni F. Mahony J. (2017). The first microbial colonizers of the human gut: composition, activities, and health implications of the infant gut microbiota. *Microbiol. Mol. Biol. Rev.* 81:e00036-17. 10.1128/MMBR.00036-17 29118049 PMC5706746

[B49] O’Connor L. E. Gahche J. J. Herrick K. A. Davis C. D. Potischman N. Vargas A. J. (2021). Nonfood prebiotic, probiotic, and synbiotic use has increased in US Adults and Children From 1999 to 2018. *Gastroenterology* 161 476–486.e3. 10.1053/j.gastro.2021.04.037. 33895169

[B50] Peh H. Y. Tan W. S. Liao W. Wong W. S. (2016). Vitamin E therapy beyond cancer: tocopherol versus tocotrienol. *Pharmacol. Ther.* 162 152–169. 10.1016/j.pharmthera.2015.12.003 26706242

[B51] Qin Y. Jiao X. Simpson J. L. Chen Z. J. (2015). Genetics of primary ovarian insufficiency: new developments and opportunities. *Hum. Reprod. Update* 21 787–808. 10.1093/humupd/dmv036 26243799 PMC4594617

[B52] Ribeiro A. E. Monteiro N. E. S. Moraes A. V. G. Costa-Paiva L. H. Pedro A. O. (2018). Can the use of probiotics in association with isoflavone improve the symptoms of genitourinary syndrome of menopause? results from a randomized controlled trial. *Menopause* 26 643–652. 10.1097/GME.0000000000001279 30531444

[B53] Rostami Dovom M. Noroozzadeh M. Mosaffa N. Zadeh-Vakili A. Piryaei A. Ramezani Tehrani F. (2019). Induced premature ovarian insufficiency by using D galactose and its effects on reproductive profiles in small laboratory animals: a systematic review. *J. Ovarian Res.* 12:96. 10.1186/s13048-019-0565-6 31619267 PMC6796372

[B54] Rudick B. J. Ingles S. A. Chung K. Stanczyk F. Z. Paulson R. J. Bendikson K. A. (2014). Influence of vitamin D levels on in vitro fertilization outcomes in donor-recipient cycles. *Fertil. Steril.* 101 447–452. 10.1016/j.fertnstert.2013.10.008 24210230

[B55] Safiyeh F. D. Mojgan M. Parviz S. Sakineh M. A. Behnaz S. O. (2021). The effect of selenium and vitamin E supplementation on anti-Mullerian hormone and antral follicle count in infertile women with occult premature ovarian insufficiency: a randomized controlled clinical trial. *Complement Ther. Med.* 56:102533. 10.1016/j.ctim.2020.102533 33197657

[B56] Samad N. Nguyen H. H. Ebeling P. R. Milat F. (2020). Musculoskeletal health in premature ovarian insufficiency. Part Two: Bone. *Semin. Reprod. Med.* 38 289–301. 10.1055/s-0041-1722849 33784746

[B57] Sapre S. Thakur R. (2014). Lifestyle and dietary factors determine age at natural menopause. *J. Midlife Health* 5 3–5. 10.4103/0976-7800.127779 24672198 PMC3955043

[B58] Scholz-Ahrens K. E. Schrezenmeir J. (2007). Inulin and oligofructose and mineral metabolism: the evidence from animal trials. *J. Nutr.* 137 2513S–2523S. 10.1093/jn/137.11.2513S 17951495

[B59] Seyed Hameed A. S. Rawat P. S. Meng X. Liu W. (2020). Biotransformation of dietary phytoestrogens by gut microbes: a review on bidirectional interaction between phytoestrogen metabolism and gut microbiota. *Biotechnol. Adv.* 43:107576. 10.1016/j.biotechadv.2020.107576 32531317

[B60] Silvestris E. Lovero D. Palmirotta R. (2019). Nutrition and female fertility: an interdependent correlation. *Front. Endocrinol.* 10:346. 10.3389/fendo.2019.00346 31231310 PMC6568019

[B61] Simpson S. Seifer D. B. Shabanova V. Lynn A. Y. Howe C. Rowe E. (2020). The association between anti-Müllerian hormone and vitamin 25(OH)D serum levels and polycystic ovarian syndrome in adolescent females. *Reprod. Biol. Endocrinol.* 18:118. 10.1186/s12958-020-00676-y 33218348 PMC7679991

[B62] Smejkal C. Kolida S. Bingham M. Gibson G. McCartney A. (2003). Probiotics and prebiotics in female health. *J. Br. Menopause Soc.* 9 69–74. 10.1258/136218003100322224 12844428

[B63] Szegda K. L. Whitcomb B. W. Purdue-Smithe A. C. Boutot M. E. Manson J. E. Hankinson S. E. (2017). Adult adiposity and risk of early menopause. *Hum. Reprod.* 32 2522–2531. 10.1093/humrep/dex304 29087465 PMC5850492

[B64] Szulińska M. Łoniewski I. van Hemert S. Sobieska M. Bogdański P. (2018). Dose-Dependent effects of multispecies probiotic supplementation on the lipopolysaccharide (LPS) level and cardiometabolic profile in obese postmenopausal women: a 12-Week randomized clinical trial. *Nutrients* 10:773. 10.3390/nu10060773 29914095 PMC6024794

[B65] Szymański W. Kazdepka-Ziemińska A. (2003). Effect of homocysteine concentration in follicular fluid on a degree of oocyte maturity. *Ginekol. Pol.* 74 1392–1396. 14669450

[B66] Taneja C. Gera S. Kim S. M. Iqbal J. Yuen T. Zaidi M. (2019). FSH-metabolic circuitry and menopause. *J. Mol. Endocrinol.* 63 R73–R80. 10.1530/JME-19-0152 31454787 PMC6992500

[B67] Tao X. Jiang A. Yin L. Li Y. Tao F. Hu H. (2015). Body mass index and age at natural menopause: a meta-analysis. *Menopause* 22 469–474. 10.1097/GME.0000000000000324 25203893

[B68] Timan P. Rojanasthien N. Manorot M. Sangdee C. Teekachunhatean S. (2014). Effect of synbiotic fermented milk on oral bioavailability of isoflavones in postmenopausal women. *Int. J. Food. Sci. Nutr.* 65 761–767. 10.3109/09637486.2014.908169 24720601

[B69] Wang J. Xu J. Han Q. Chu W. Lu G. Chan W. Y. (2020). Changes in the vaginal microbiota associated with primary ovarian failure. *BMC Microbiol.* 20:230. 10.1186/s12866-020-01918-0 32727366 PMC7392721

[B70] Wang L. Tang J. Wang L. Tan F. Song H. Zhou J. (2021). Oxidative stress in oocyte aging and female reproduction. *J. Cell Physiol.* 236 7966–7983. 10.1002/jcp.30468 34121193

[B71] Wang M. Gong W. W. Hu R. Y. Wang H. Guo Y. Bian Z. (2018). Age at natural menopause and associated factors in adult women: findings from the China Kadoorie Biobank study in Zhejiang rural area. *PLoS One* 13:e0195658. 10.1371/journal.pone.0195658 29668705 PMC5905992

[B72] Weaver C. M. Martin B. R. Nakatsu C. H. Armstrong A. P. Clavijo A. McCabe L. D. (2011). Galactooligosaccharides improve mineral absorption and bone properties in growing rats through gut fermentation. *J. Agric Food Chem.* 59 6501–6510. 10.1021/jf2009777 21553845

[B73] Wei J. Chen J. R. Pais E. M. A. Wang T. Y. Miao L. Li L. (2017). Oxyresveratrol is a phytoestrogen exerting anti-inflammatory effects through NF-κB and estrogen receptor signaling. *Inflammation* 40 1285–1296. 10.1007/s10753-017-0572-y 28484893

[B74] Westphal L. M. Polan M. L. Trant A. S. (2006). Double-blind, placebo-controlled study of fertilityblend: a nutritional supplement for improving fertility in women. *Clin. Exp. Obstet Gynecol.* 33 205–208. 17211965

[B75] Wu X. Kim M. J. Yang H. J. Park S. (2021). Chitosan alleviated menopausal symptoms and modulated the gut microbiota in estrogen-deficient rats. *Eur. J. Nutr.* 60 1907–1919. 10.1007/s00394-020-02382-2 32910260

[B76] Wu Y. Y. Liang C. Y. Liu T. T. Liang Y. M. Li S. J. Lu Y. Y. (2018). Protective roles and mechanisms of polysaccharides from dendrobium officinal on natural aging-induced premature ovarian failure. *Biomed. Pharmacother.* 101 953–960. 10.1016/j.biopha.2018.03.030 29635905

[B77] Xu J. Hennebold J. D. Seifer D. B. (2016). Direct vitamin D3 actions on rhesus macaque follicles in three-dimensional culture: assessment of follicle survival, growth, steroid, and antimüllerian hormone production. *Fertil. Steril.* 106 1815–1820.e1. 10.1016/j.fertnstert.2016.08.037. 27678030 PMC5136302

[B78] Yang L. C. Wu J. B. Lu T. J. Lin W. C. (2013). The prebiotic effect of *Anoectochilus formosanus* and its consequences on bone health. *Br. J. Nutr.* 109 1779–1788. 10.1017/S0007114512003777 22950799

[B79] Ye L. Cao Z. Lai X. Wang W. Guo Z. Yan L. (2019). Niacin fine-tunes energy homeostasis through canonical GPR109A signaling. *FASEB J.* 33 4765–4779. 10.1096/fj.201801951R 30596513

[B80] Yoneda T. Ueno T. Uchiyama S. (2011). S-equol and the fermented soy product SE5-OH containing S-equol similarly decrease ovariectomy-induced increase in rat tail skin temperature in an animal model of hot flushes. *Menopause* 18 814–820. 10.1097/gme.0b013e318208fb0d 21451423

